# Feature Extraction for Dimensionality Reduction in Cellular Networks Performance Analysis

**DOI:** 10.3390/s20236944

**Published:** 2020-12-04

**Authors:** Isabel de-la-Bandera, David Palacios, Jessica Mendoza, Raquel Barco

**Affiliations:** 1 Department of Communications Engineering, University of Málaga, 29071 Málaga, Spain; jmr@ic.uma.es (J.M.); rbm@ic.uma.es (R.B.); 2 Tupl Spain S.L., Tupl Inc., 29010 Málaga, Spain; david.palacios@tupl.com

**Keywords:** dimensionality reduction, feature extraction, mobile networks

## Abstract

Next-generation mobile communications networks will have to cope with an extraordinary amount and variety of network performance indicators, causing an increase in the storage needs of the network databases and the degradation of the management functions due to the high-dimensionality of every network observation. In this paper, different techniques for feature extraction are described and proposed as a means for reducing this high dimensionality, to be integrated as an intermediate stage between the monitoring of the network performance indicators and their usage in mobile networks’ management functions. Results using a dataset gathered from a live cellular network show the benefits of this approach, in terms both of storage savings and subsequent management function improvements.

## 1. Introduction

Management tasks in current mobile networks, such as network optimization or fault management, have become cumbersome and expensive, due to the high complexity of modern networks, thus increasing both the operational and the capital expenditure (OPEX and CAPEX, respectively). In order to overcome this, the automation of such management tasks has become an important challenge, which is even more relevant for 5G networks [1]. However, the huge amount of available data related to different services and network segments decreases the efficiency of the automation of such management functions due to the fact that storage and algorithms are not prepared to work with such big data. To know the current network state (e.g., whether the network behavior is sub-optimal or degraded), these management functions rely on network performance indicators, which in the context of cellular networks are called key performance indicators (KPI). These indicators quantify the performance of network processes and functionalities and are monitored and stored by the Operations Support System (OSS) of the cellular network. As a result, and for network management tasks to be optimally performed, Mobile Network Operators (MNOs) and vendors have put a lot of effort into arranging a sufficiently detailed and varied amount of KPIs. However, many of these KPIs do not provide relevant information about the network state. The result is a large amount of data that has to be stored in network nodes, causing storage problems. Moreover, such amount of data may produce over-fitting of the algorithms used for the automation of the management tasks. To avoid these problems, an efficient selection of a set of KPIs should be carried out in order to optimize the network management tasks. Traditionally, troubleshooting experts have made this selection manually [2], being a task that is quite time-consuming and highly complex in nature. These limitations have caused many experts to use the same set of KPIs, the ones that usually have shown better results in terms of error metrics, in most of the management problems. However, the set of KPIs that is traditionally selected could not be the most suitable one for some specific management tasks, leading to non-optimal performance. To cope with this issue, dimensionality reduction has become an essential preprocessing task.

Regarding cellular network management, few works have been reported in this field. Some works have applied feature selection techniques with the aim of classifying traffic flows. For example, in [3], a supervised correlation-based technique for KPI selection is applied in a UMTS (Universal Mobile Telecommunications System) network. In [4], however, authors applied dimensionality reduction to improve the classification of Internet traffic. In [5,6], supervised techniques for automatic feature selection are applied to determine the most useful KPIs to eventually identify the cause for a performance degradation in a cellular network. Specifically, in [5], a supervised technique based on a genetic algorithm is proposed. The authors of [6] also propose a supervised method, which relies on the statistical dissimilarity of KPIs when conditioned to different network states.

However, given their supervised nature, the works in [5,6] rely on the availability of a network status label attached to every network observation, which seldom is present. Actually, most of the performance management data stored in nowadays cellular networks are unlabeled, due to the high amount of time required for network experts to analyze and document every network fault, together with the pressing need for a fast response. In this case, an unsupervised selection method can be applied for dimensionality reduction which carries out a selection of a representative set of KPIs. Specifically, in [7], an unsupervised technique for KPI selection is proposed as a clustering module followed by a supervised feature selection technique.

In addition to feature selection, dimensionality reduction encompasses a second family of Machine Learning techniques, which is feature extraction. Feature extraction techniques convert the original features into a new and lower-dimensional set of synthetic features. The application of feature extraction to cellular network management is presented in several works. The authors of [8] propose a framework for Self-Organizing Network (SON) coordination where dimensionality reduction stage is implemented by using Self-Organizing Maps (SOM). In [9], different techniques for dimensionality reduction are applied to time series prediction. Finally, Ref. [10] is focused on the combination of selection and extraction techniques as part of a fault management framework.

A possible limitation of the application of dimensionality reduction methods to management tasks is a reduction in the accuracy of the results. Regarding this issue, a trade-off between accuracy and storage needs must be achieved. These types of decisions are common in autonomic management problems [11].

This paper presents a detailed comparison among different feature extraction techniques and their application to a fault diagnosis problem. Unlike [10], which is more focused on feature selection techniques and where only one feature extraction technique is considered, this work evaluates the advantages of using different feature extraction methods to improve fault diagnosis results. In addition, although the work presented in [9] is also based on feature extraction techniques, the problem addressed is highly different to the one considered in this work. Feature extraction techniques are a set of methods that allow for reducing the dimensionality of problems of different nature. These methods select a small group of features from a large dataset to combine them and compute new features. These new features contain the most important information from the original dataset regarding the scenario where this technique is being applied. Therefore, the considered problem for the application of feature extraction has an important impact on the final result. The objective of this work is to analyze the suitability of the mostly used families of techniques for feature extraction when they are applied to a fault diagnosis problem in a cellular network. The use of these techniques may not only reduce the dimensionality of the diagnosis problem, but also improve the fault diagnosis results. With this aim, the diagnosis error rate (DER) indicator is used, which stands for the samples’ misclassification rate. In addition to DER, the data storage needs are also considered as a key metric for the evaluation of each technique.

The rest of the paper is organized as follows: firstly, the problem formulation is presented, describing the scenario considered in this work. Secondly, an overview of feature extraction techniques is described, focusing on two specific feature extraction families. Then, the performance analysis is shown. Details of the experiments carried out and obtained results are provided. Finally, some conclusions are drawn from the previous study and tests.

## 2. Problem Formulation

**Figure 1:**
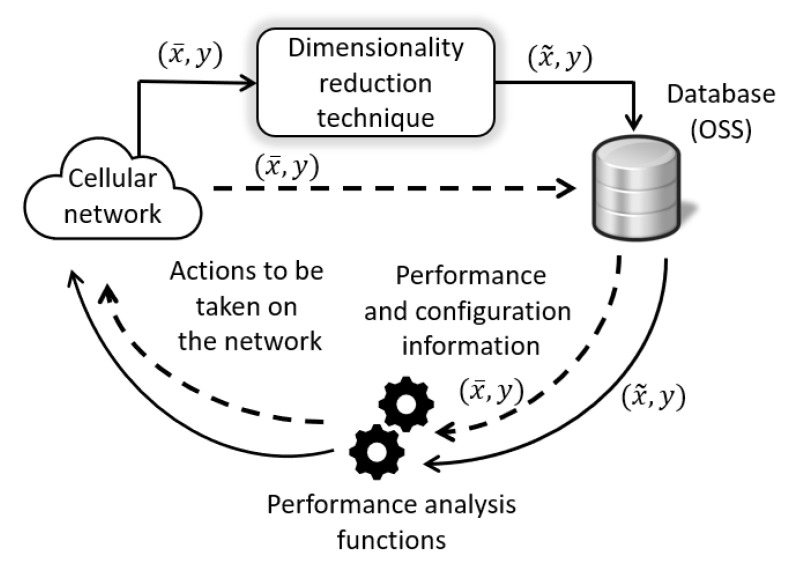
Comparison of a typical (dashed lines) and proposed (solid lines) schemes for network performance information gathering and use in performance analysis functions.

In a cellular network, performance information is monitored and periodically stored in a centralized database: the OSS database (see dashed lines in Figure 1). This information usually takes the shape of service or network performance metrics (such as KPIs), as well as user call traces, and may be expressed as a set of *N*-dimensional samples, $\bar{x}=\left\{x_{1},x_{2},\ldots ,x_{N}\right\}$, being *N* the number of the monitored metrics, that is, the number of features. A feature could be, for example, the number of user connection attempts registered by a base station in a given time period. Usual values for *N* (several thousands), together with the high number of network elements simultaneously monitored, entail the storage of a huge amount of performance data every day and pose a performance issue for subsequent fault diagnosis functions. Together with $\bar{x}$, a possible additional label, *y*, often referred to as the ground truth label, may be attached. This label corresponds to the network state under which each sample was gathered. A ground truth label could be a coverage hole, a problem of interference, or a network overload. The availability of this label allows using supervised techniques for dimensionality reduction [3,5] and supervised techniques for automatic diagnosis [12,13], whereas its lack forces network experts to only use unsupervised techniques for both dimensionality reduction and RCA functions [2].

In the context of performance analysis, diagnosis functions take the shape of classifying systems, Figure 1, also called automatic diagnosis systems. Specifically, in a classification problem, a dimensionality reduction technique is a procedure that, before the classification itself, either finds or generates a set of *q* features (with q<<N) that represents a sample with the minimum loss of useful information compared to the original set. That is, after dimensionality reduction has taken place, the classifier takes $\tilde{x}\in R^{q}$ as its input, instead of $\bar{x}\in R^{N}$ (see solid lines in Figure 1).

## 3. Feature Extraction

Feature extraction is a family of dimensionality reduction techniques where a new set of features is built from the original feature set. In order to reduce dimensionality, the number of the new features is lower than the number of the original ones. Feature extraction is a tool that projects the original features onto a more convenient and reduced basis. These new features are computed in such a way that they retain as much information as possible from the original feature set.

**Figure 2:**
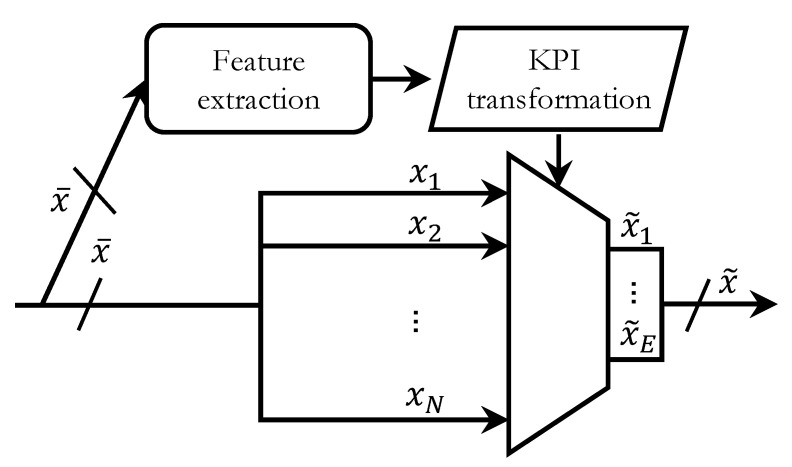
Scheme for feature extraction in the context of fault diagnosis functions. The upper branch represents the model creation during the learning phase (in this case, the KPI transformation to be applied), whereas the lower branch represents the application of such KPI transformation.

In the context of cellular networks, this means that a new set of synthetic KPIs are built upon the combination of the original ones retaining as much information as possible. Therefore, a feature extraction technique is a process that transforms an element $\bar{x}\in R^{N}$, defined by the features $\left\{x_{1},\ldots ,x_{N}\right\}$, in an element $\tilde{x}\in R^{q}$, defined by the features $\left\{{\tilde{x}}_{1},\ldots ,{\tilde{x}}_{q}\right\}$, where each new feature ${\tilde{x}}_{j}$ comes from the combination of several features of $\bar{x}$. Even though $\tilde{x}$ may have any number of dimensions, in general, it is preferred that q<N, thus making $\tilde{x}$ contain the information of $\bar{x}$ in a lower number of dimensions. A scheme for feature extraction is shown in Figure 2. In this case, given that the vast majority of feature extraction techniques is unsupervised, only $\bar{x}$ is used as its input. The result is a model for KPI transformation that may differ depending on the particular technique being used. For example, it may consist of a matrix describing how the indicators at the input are linearly combined to produce the synthetic ones. It may also describe nonlinear functions to be applied over the former to get the latter. In any case, the resulting output takes the form of $\tilde{x}$: a vector with less dimensions that $\bar{x}$ and which is comprised of features ${\tilde{x}}_{1}$ to ${\tilde{x}}_{q}$. The tilde in ${\tilde{x}}_{1}$ to ${\tilde{x}}_{q}$ highlights the fact that these features are different from those at the input, not being a subset of the latter, such as would be the case in feature selection techniques.

The reason why feature extraction techniques get to retain a higher amount of useful information than feature selection techniques given the same number of resulting features is that not all the information retained in the *selected* (contrary to the *extracted*) KPIs is *useful* information. This fact is mainly due to the nature of the magnitude being quantified and its suitability to represent the performance of the underlying process. At this point, the utility of a feature, or KPI, can be quantified as its variance with respect to the variance of the whole dataset, considering all the KPIs. Often, the techniques for feature extraction have as the criterion that the per-feature variance is maximized, thus reducing the number of necessary features to retain the whole variance. This is the case for the techniques based on principal component analysis (PCA) [14].

This work is focused on the application of two of the most promising feature extraction families to cellular network performance analysis. The application methodology followed in this work for all the feature extraction techniques is the same. That is, in all cases, the selected feature extraction technique is applied before a fault diagnosis stage. As a result of this application, a set of KPIs from the available dataset is selected in order to compute some synthetic KPIs. Therefore, these synthetic KPIs are obtained as a combination of the selected KPIs. Each technique obtains a different set of synthetic KPIs since the learning process is different for each method. More details about the functioning of the feature extraction techniques and their learning processes can be found in the references associated with each technique. These new KPIs are the ones used in the subsequent stage of fault diagnosis. Based on the results obtained in the diagnosis stage, it is possible to determine the most appropriate feature extraction technique to be used in a fault management problem.

### 3.1. Component Analysis

The techniques based on component analysis define the features transformation using information from their statistical behavior. Within this group, one may find techniques implying both a linear transformation over the original features (e.g., PCA) and techniques implying nonlinear transformations, like the kernel PCA (kPCA).

- **PCA:** Principal component analysis is one of the most used feature extraction techniques in a wide variety of fields of science, due to its high effectiveness and ease of implementation. In the context of mobile networks, given an original set of KPIs of *N* dimensions, PCA determines the *q* dimensions or hyperplanes that, being orthogonal among them and a linear combination of the former *N* KPIs, maximize the variance of the projection of the original samples.

**Figure 3:**
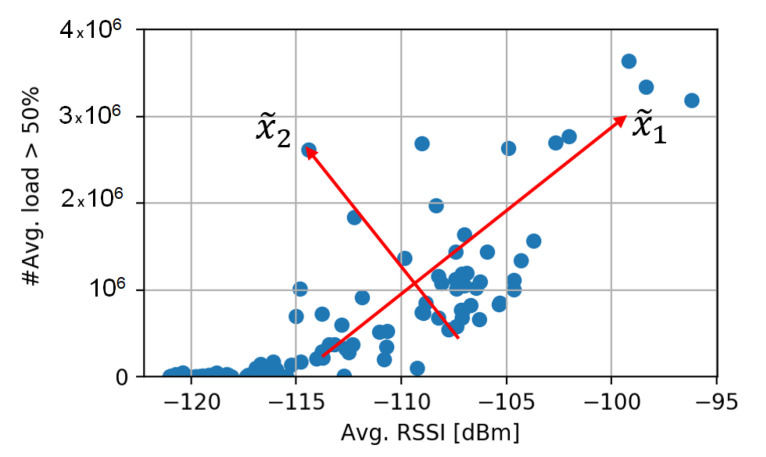
Resulting features of the application of PCA (red lines, $\tilde{x}$) over a set of samples of two KPIs.

Figure 3 shows, as an example, the resulting features of applying PCA over a set of test KPIs. The orthogonality of ${\tilde{x}}_{1}$ and ${\tilde{x}}_{2}$ can be seen as a result of applying PCA.

- **Kernel PCA (kPCA):** this nonlinear feature extraction technique applies PCA over the KPIs resulting from a nonlinear transformation of the original KPIs [15]. The nonlinear function applied on the original space is known as the *kernel*. This way, simple hyperplanes, resulting from the application of PCA, defined over the transformed space, result in complex structures in the space of the original KPIs.
- **Independent component analysis (ICA):** Unlike PCA, which only looks for the orthogonality of the resulting synthetic KPIs, ICA targets the statistic independence of these—generally by means of minimizing the mutual information among the features [16]. Linear and nonlinear variants of this technique exist.

### 3.2. Manifold Learning

The methods based on manifold learning are a set of nonlinear techniques for feature extraction. They rely on the premise that a set of samples of high dimensionality is indeed a body with a set of a lower number of dimensions, whose shape has been manipulated to result in the latter. This can be seen as a 2D plane which has been rolled up to result in a three-dimensional structure. In a problem of classification, such as a fault diagnosis based on KPIs, it would be easier to define the decision boundaries over a two-dimensional structure instead of over a three-dimensional structure.

**Figure 4:**
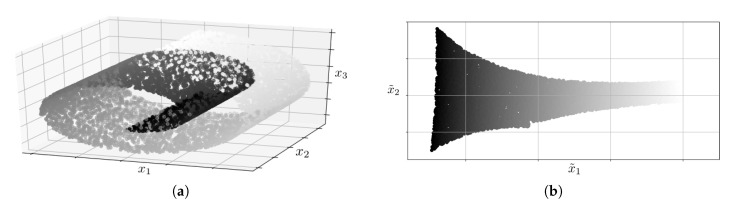
Example of feature extraction using LLE, a technique based on manifold learning, from [17]. (**a**) samples $\bar{x}$ in the *N*-dimensional original space; (**b**) samples $\tilde{x}$ in the *q*-dimensional transformed space.

Figure 4 shows an example of this. In Figure 4a, a 2D plane which has been rolled up to result in a three-dimensional structure is depicted. Figure 4b shows the result of unwrapping the plane after applying a manifold learning technique. In the problem of classification, it would be difficult to define the decision boundaries over a structure as the one shown in Figure 4a; however, it would be easy to do this with the structure shown in Figure 4b.

Some of the most well-known feature extraction techniques based on manifold learning are the following:

- **Locally-linear embedding, LLE:** this technique allows determining the *q*-dimensional space which better preserves the distance between the projection of each KPI and their neighbors’ [18]. Figure 4 shows an example of the application of this technique.
- **Spectral embedding, SE:** this technique, like LLE, uses the concept of neighborhood among KPIs, in this case, to define the graph whose spectral decomposition allows defining the *q*-dimensional space onto which projecting the original space [19].

## 4. Performance Analysis

In order to show the feasibility of feature extraction techniques for cellular network management, in this section, the presented methods have been assessed regarding their ability to reduce the number of features at the input of a method for fault diagnosis while preserving as much useful information as possible.

### 4.1. Experiment Setup

A test has been carried out to evaluate the performance of the considered feature extraction techniques in the field of fault diagnosis. In the experiments, a dataset from a live LTE network with 359 samples has been used [20]. The methodology and analysis proposed in this paper can be easily applied to a 5G network when a proper dataset is available. Each sample is composed of 286 RAN KPIs and a ground truth label, indicating the network state under which the sample was collected. In particular, four different labels are differentiated: high traffic, no traffic, high CPU utilization, and low coverage. As described before, the comparison among the selected feature extraction algorithms is made by evaluating the DER obtained in a subsequent diagnosis stage. The inputs used in the diagnosis stage are the synthetic KPIs computed by each feature extraction technique. DER is defined as the ratio of problematic cases diagnosed with a different label from the real one (misclassified cases), $N_{MPC}$, to the total number of problematic cases, $N_{PC}$, as shown in the following expression:

(1) $$DER=\frac{N_{MPC}}{N_{PC}}$$

An LDA (linear discriminant analysis) classifier is used as the diagnosis tool. Since the feature extraction techniques do not need the ground truth label for their application, the database is treated as unlabeled during this procedure. Therefore, labels from the dataset are only used in order to compute the DER, as a means to quantify the validity of the chosen indicators. Regarding the available KPIs, they provide information about mobility, accessibility, CPU load, retainability, and throughput.

Seven different situations for feature extraction are distinguished. First, to set a baseline, all the KPIs are used, representing the situation when no selection is performed. Then, four techniques based on component analysis have been assessed: PCA, kPCA using a Gaussian kernel (named kPCA${}_{1}$ onwards), kPCA using a sigmoid kernel (named kPCA${}_{2}$ onwards) and ICA. Finally, two feature extraction techniques based on manifold learning are used: LLE and SE. The same number of synthetic KPIs are considered in all these situations, 10.

The dataset has been partitioned following a 0.4, 0.4, 0.2 split, devised for the computation of the KPI transformation model for the feature extraction techniques, the training of the LDA classifier, and the testing of this classifier, respectively. Given that the dataset considered in this work is not big enough, the variance of the results is high. Therefore, the conclusions that can be obtained are not reliable. One approach to reduce this variance is the application of repetitions in the experiments. With the aim of obtaining more reliable results when the number of cases is scarce either in the testing or in the modeling set, a stratified Monte Carlo cross-validation (MCCV) of 50 repetitions has been performed per each step of the modeling-to-testing ratio. That is, the samples assigned to each set have been randomized in each repetition preserving the relative frequency of each cause in these subsets. Then, the resulting DERs have been averaged over the 50 repetitions. Finally, a standard normalization is applied to each split. The selection of 50 repetitions is a result of a trade-off between a reduced number of repetitions, in order to limit the computational load of the approach, and the reliability of the results. Some experiments have been carried out in order to find the appropriate number of repetitions. In these experiments, different numbers of repetitions have been tested until the variance of the results was low enough.

### 4.2. Results and Discussion

**Figure 5:**
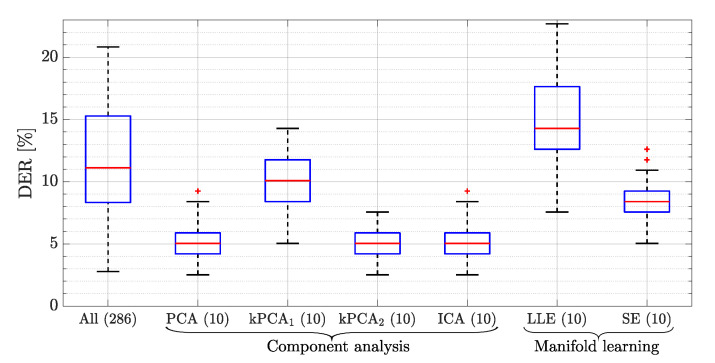
Diagnosis error rate (DER) of diagnosis system based on LDA for different feature extraction families and techniques. Crosses are included representing the outliers.

**Figure 6:**
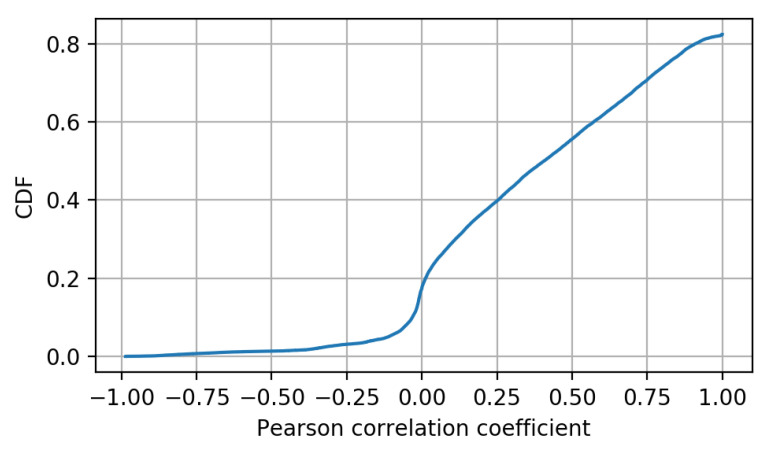
Cumulative Distribution Function of Pearson correlation coefficient obtained for the set of KPIs.

Figure 5 shows the resulting DERs for this test. Each box plot represents the first, second, and third quartile ($Q_{1}$, $Q_{2}$ and $Q_{3}$, respectively; from the bottom, the blue, the red, and the next blue horizontal lines) as well as the lower and upper adjacent values (i.e., $Q_{1}-1.5\cdot \left(Q_{3}-Q_{1}\right)$ and $Q_{3}+1.5\cdot \left(Q_{3}-Q_{1}\right)$, respectively) for each method throughout the 50 iterations. Outliers are shown as crosses. In light of this, all the component analysis-based techniques except for kPCA${}_{1}$ provide a lower DER than the case with all the KPIs. The reason for the good results of PCA comes from the nature of the KPIs being monitored and the relation they have among each other, being fundamentally linear. In turn, this linearity appears from the nature of the processes being quantified, this being a common issue in cellular networks. The process to obtain KPIs from a network consists of the application of different formulas for the combination of performance counters. These performance counters are metrics gathered by the different nodes of the network and are related to the different procedures that are taking place. Some examples of these counters are the number of connection attempts or the number of successful connections. Based on these metrics, KPIs such as the ratio of successful connections can be obtained. Thus, KPIs that are obtained based on similar performance counters can present a high linear relation. It is also possible to find KPIs that are not related to other KPIs. For example, a KPI related to cell availability (meaning the time during an hour that the cell is available) is not related to any other KPI. However, most of the common KPIs are related to at least other KPI in a linear manner. To confirm this behavior, Pearson correlation coefficients have been obtained and represented by means of their cumulative density function, Figure 6. Specifically, the correlation for every pair of KPIs from the considered dataset has been obtained. It can be seen that more than 50% of the obtained coefficients are above 0.5, indicating a high level of linearity among KPIs.

In the case of kPCA${}_{2}$, the normalization of the KPIs and the distribution of many of them around zero makes the sigmoid kernel approximate to a linear function, providing an optimum final DER, similar to the one provided by PCA. On the other hand, the resulting DERs of both kPCA${}_{1}$ and the techniques based on manifold learning provide poorer results, even worse than the baseline case for LLE. The reason is that these techniques assume a strong nonlinearity in the dataset, which is contrary to the linear character of the relations among the considered KPIs.

As described before, the amount of data that can be collected from a mobile network is very large and it is increasing with the arrival of new generations of mobile technologies. Based on this large amount of information, experts must analyze the performance of the network and not only detect if the network behavior is normal, but also identify the specific problem that is affecting a specific area of the network. Moreover, when the decision is based on real data, it is important to consider that part of this information may affect the accuracy of the classification negatively. This fact is shown in the baseline results, obtaining a worse DER when all the available information is used to make the fault classification.

Based on these results, the application of feature extraction techniques to a mobile network management allows for reducing the storage needs up to 96% (using 10 KPIs instead of all 286 monitored KPIs) while improving the DER of a subsequent diagnosis stage more than 50%. The possible limitations of this approach are a possible reduction in the accuracy of the results, an increase in the complexity of the system and its adaptation to changes in the network. In relation to the former, it is important to take into account that an extremely high reduction may reduce the accuracy of the diagnosis result. In [10], the relation between the DER and the number of computed synthetic counters from a PCA method is shown. In addition, the complexity of the feature extraction method needs to be considered in the whole fault management framework when it is applied to a network. Regarding the adaptation of changes in the network, it represents the main limitation of this approach. When the feature extraction method is applied to a network, the main KPIs to perform the fault diagnosis are determined in order to compute the synthetic KPIs that will be used in the fault diagnosis stage. This selection is specific for the concrete faults that are being considered. If new faults appear in the network, it will be necessary to perform the training of the feature extraction method again in order to adapt the KPI selection including these new faults. One possible approach could be to perform this training periodically, so that the feature extraction method would be adapted to network changes.

## 5. Conclusions

This paper has presented the assessment of different techniques for feature extraction, to be integrated as an intermediate stage between the monitoring of the network KPIs and their usage in performance analysis in a mobile network. At the expense of losing the meaning of the resulting KPIs, feature extraction techniques allow for condensing relevant performance information in a more reduced set of synthetic KPIs. The results of using a set of data collected from a live cellular network have shown the benefits of this approach in terms of storage savings and subsequent improvements to the fault diagnosis function. The benefits have been found to be specially relevant when linear techniques for feature extraction are used, given the mostly linear dependence of the most common KPIs.
